# Control of Pierce's Disease by Phage

**DOI:** 10.1371/journal.pone.0128902

**Published:** 2015-06-24

**Authors:** Mayukh Das, Tushar Suvra Bhowmick, Stephen J. Ahern, Ry Young, Carlos F. Gonzalez

**Affiliations:** 1 Department of Plant Pathology and Microbiology, Texas A&M University, College Station, Texas, United States of America; 2 Center for Phage Technology, Texas A&M University, College Station, Texas, United States of America; 3 Department of Biochemistry and Biophysics, Texas A&M University, College Station, Texas, United States of America; University of Idaho, UNITED STATES

## Abstract

Pierce’s Disease (PD) of grapevines, caused by *Xylella fastidiosa* subsp. *fastidiosa* (*Xf*), is a limiting factor in the cultivation of grapevines in the US. There are presently no effective control methods to prevent or treat PD. The therapeutic and prophylactic efficacy of a phage cocktail composed of four virulent (lytic) phages was evaluated for control of PD. *Xf* levels in grapevines were significantly reduced in therapeutically or prophylactically treated grapevines. PD symptoms ceased to progress one week post-therapeutic treatment and symptoms were not observed in prophylactically treated grapevines. Cocktail phage levels increased in grapevines in the presence of the host. No *in planta* phage-resistant *Xf *isolates were obtained. Moreover, *Xf* mutants selected for phage resistance *in vitro* did not cause PD symptoms. Our results indicate that phages have great potential for biocontrol of PD and other economically important diseases caused by *Xylella*.

## Introduction

Pierce's Disease (PD) caused by the xylem-limited, gamma-proteobacterium *Xylella fastidiosa* subsp. *fastidiosa* (*Xf*) [1], is a severe threat to the wine industry in the United States. *Xf* is transmitted by xylem sap-feeding insect vectors and colonizes the xylem vessels of host plants by movement through bordered pits to neighboring vessels. The formation of biofilms within the vessels results in plugging and disease [2]. Existing disease control methods are often only partially successful. Other than rouging of infected grapevines, the most widely used current practice relies on controlling the vectors through widespread application of systemic insecticides such as neonicotinoids that are absorbed by plants and transferred throughout the vascular system [3,4]. Neonicotinoids are neuro-active insecticides that derive their toxicity by acting mainly agonistically on nicotinic acetylcholine receptors on the post-synaptic membrane [5,6,7]. The neonicotinoid pesticide, imidacloprid, is one of the most widely used insecticides in the world and is recommended for the control of the glassy winged sharpshooter (*Homalodisca coagulate*), a major vector of *Xf* [3,5]. However, recent concerns of the role of neonicotinoids in honey bee colony collapse disorder [8] has resulted in a two year ban of three neonicotinoid insecticides (clothianidin, imidacloprid and thiamethoxam) by the European Commission because of the acute and chronic effects on bee colony survival and development [9]. In 2013, the US Environmental Protection Agency issued a notification to registrants on registered pesticides containing imidacloprid, dinotefuran, clothianidin or thiamethoxam advising of strengthen pollinator protective labeling on neonicotinoid products [10].

Clearly there is a need to develop alternative methods for control PD. Current approaches include the development of transgenic grapevines and rootstocks resistant to PD [11,12,13,14] and the use of avirulent *Xf* strains to protect susceptible grapevines [15]. We submit that the used of bacteriophages (phages) offers an environmentally-friendly, effective, and sustainable biocontrol method for PD.

Phages are the most abundant and ubiquitous genetic entity on earth [16,17]. Besides ubiquity, two major properties of phages, specificity and exponential propagation, make them attractive as antibacterial agents. Specifically, a cocktail of phages exhibiting broad host range activity that reflects a diversity of receptors would maximize the potency of the treatment and minimize the possibility for development of resistance. Well-developed phage cocktails can act as a natural biocontrol for bacterial infections, targeting a pathogenic bacterium without affecting the animal or plant host or their commensal microflora. Guidelines to the development of phage-based biocontrol strategies are yet to be formalized but it is already generally accepted that temperate phages should not be candidates for therapeutic use because of their potential capacity for specialized or generalized transduction of pathogenesis determinants [18,19].

In this study, a cocktail of phages previously shown to be incapable of lysogeny [20] was evaluated for therapeutic and prophylactic treatment of PD in *Vitis vinifera* in greenhouse studies. Considering the practical application of the phage biocontrol system, we also addressed the potential for the development of phage-resistant mutants. This is the first report for the use of phages to treat or prevent PD of grapevines.

## Materials and Methods

### Bacterial strains, phages and inoculum preparation

All bacterial strains used in this study are listed in S1 Table. *Xf* strain Temecula 1 (*Xf*-T1) was used in greenhouse experiments. 50 *Xf* isolates obtained from grapevine (*V*. *vinifera*) samples expressing PD symptoms from vineyards in Santa Clara, Napa, Sonoma counties, CA and Uvalde County, TX were used in molecular typing and phage host range testing (S1 Table). Cultures were maintained on PW-M agar (PW-MA) medium as described by Ahern *et al*. (2014) [20,21]. For grapevine inoculations, five-day-old cultures of the *Xf* isolates were used to make bacterial suspensions in P-buffer [15]. Phages Sano, Salvo, Prado and Paz were used in this study [20]. High-titer phage lysates (10^10^ PFU/ml) were prepared and titered as described by Ahern *et al*. (2014) [20]. Lysates were stored at 4°C.

### Ethics Statement

A United States Department of Agriculture-Animal and Plant Health Inspection Service (USDA-APHIS) Permit was issued for interstate transfer of isolates and/or plant material collected in locations listed in S1 Table. Transfer of bacterial isolates and plant material was authorized by USDA-APHIS permit P526P-11-02320. California isolates were collected on land owned or leased by Ridge Vineyards in Santa Clara, Napa and Sonoma counties, CA. Texas isolates were collected on land in Uvalde County, TX owned by the Texas A&M AgriLife Research and Extension Service. No endangered or protected species were sampled. No animals were involved in this study.

### Phage host range and host simple sequence repeat (SSR) analysis

SSR analysis was performed to identify the genetic diversity within the same 50 *Xf* isolates (S1 Table) using 11 of the SSR loci described by Lin *et al*. (2005) [22]. *Xf*-T1, *X*. *fastidiosa* subsp. *sandyi* (strain Ann-1) and *X*. *fastidiosa* subsp. *multiplex* (strain Dixon) were also used for SSR analysis. The host range of cocktail phages was determined by the serial dilution spot assay on overlays as described by Ahern *et al*. [20] using the panel of 50 *Xf* isolates as hosts.

### Grapevine growth conditions

**Fig 1:**
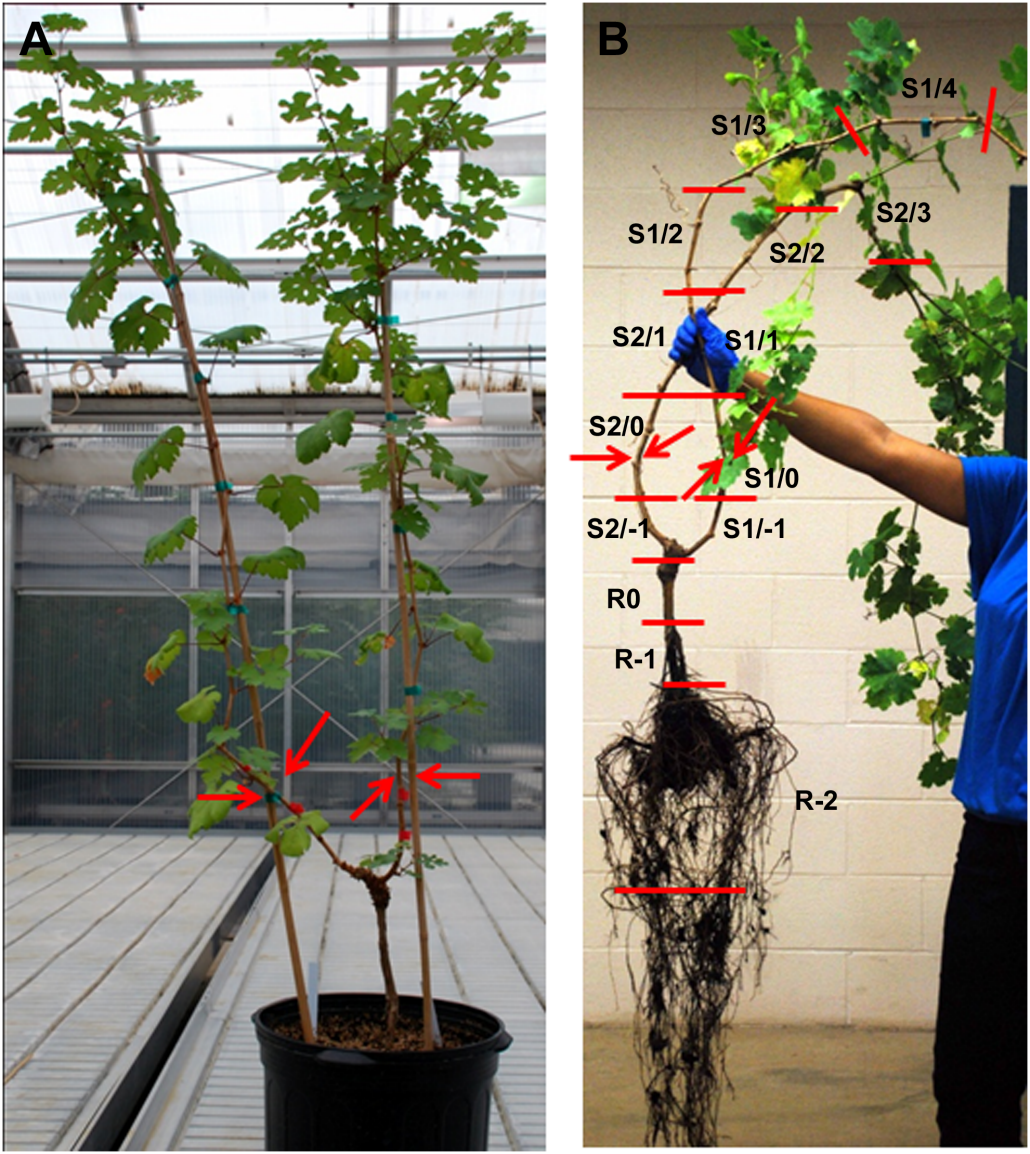
Grapevines, points of inoculation and harvest method. (A) Trained grapevine exhibiting two cordons. POI indicated with red arrows. (B) Grapevine root, trunk and cordon segments were numbered from POI (0), below (-) or above (+) in ~13 cm segments. Root portion was divided into three segments and numbered as R0, R-1, or R-2.

Dormant *V*. *vinifera* cv. Cabernet Sauvignon clone 08 on 1103P rootstock were purchased from Vintage Nurseries (Wasco, California, USA). Grapevines were planted in 7-gallon pots using 101 Sunshine Mix 1 (Sun Gro Horticulture, Vancouver, British Columbia, Canada). Grapevines were grown in a greenhouse on a 16 h light (26°C, 300–400 μE m^-2^ s^-1^)/8 h dark (18°C) cycle supplemented with illumination from sodium vapor lamps. Grapevines were watered every other day with tap water and fertilized with Peter's General Purpose 20-20-20 fertilizer and micronutrients every 15 d. Grapevines were progressively pruned to provide uniform plants with two unbranched solitary shoots. Lateral shoots and buds were removed. For each grapevine, two cordons were staked and allowed to grow until each cordon was ~80 cm in length before grapevines were used for experiments (Fig 1A).

### Grapevine inoculation with bacteria and phage

Three independent experiments in triplicate were conducted to determine phage efficacy using identical protocols. One-way analyses of variance (ANOVAs) were performed as described below. No significant difference (*P* > 0.05) was found among the three experiments using the above analysis, we therefore report the results from a single representative trial. The trial was conducted using a phage cocktail (Sano, Salvo, Prado and Paz) to evaluate phage therapeutic and prophylactic efficacy. Experimental design is described below and summarized in S1 Fig.

For disease control and to evaluate pathogen movement, 9 grapevines inoculated with *Xf*-T1 were assayed in triplicate immediately after inoculation (0 min) and at weeks 8, and 12 post-pathogen inoculation (S1 Fig, Column 2). Individual cordons were inoculated between the second and the third node on opposite sites (two points/cordon) with 40 μl of bacterial suspension (1 × 10^9^ CFU/ml) using the needle inoculation technique as described by Hopkins (2005) [15] (Fig 1A). Control grapevines were mock inoculated with P-buffer using the same protocol (S1 Fig, Column 3). Grapevines were not watered 4 d prior inoculation to insure uptake of the inoculation.

To determine phage distribution and the effect of the phage cocktail on grapevines, 21 grapevines were treated with cocktail (1 × 10^10^ PFU/ml) as described above (S1 Fig, Column 4). Grapevines treated only with phage were assayed in triplicate immediately after treatment and at every two weeks for 12 weeks.

In the therapeutic phage treatment, 15 grapevines were inoculated with *Xf*-T1 as above (S1 Fig, Column 5). At week 3 post-pathogen inoculation, *Xf*-T1 inoculated 15 grapevines were treated with 40 μl of a phage cocktail suspension (1 × 10^10^ PFU/ml) using the same inoculation protocol and technique as above. *Xf* and phage levels were evaluated (in triplicate grapevines) at the time of inoculation (0 min), 6, 8, 10 and 12 weeks, as described below.

In the prophylactic phage treatment, 15 grapevines were treated with phage cocktail using the same inoculation protocol as above, and at week 3 post-phage treatment grapevines were inoculated with *Xf*-T1 as above (S1 Fig, Column 6). Grapevines were assayed in triplicate as described below immediately after treatment (0 min) and at weeks 6, 8, 10, and 12 post treatment. All grapevines were evaluated for disease symptom development twice weekly for 12 weeks. Grapevines were scored as positive for PD symptoms when a single leaf exhibited symptoms. Grapevines inoculated with P-buffer were assayed to confirm the absence of *Xf* and/or phage by quantitative real time PCR (qRT-PCR), as described below.

### Sample collection and processing

Grapevine samples were designated, collected and processed as shown in Fig 1B. Briefly, duplicate cordons and root system from each grapevine were excised from the trunk and divided into 5–6 segments (~13 cm each). Each segment was homogenized using a 20 × 115 mm generator (PRO Scientific, CT, USA) in 15 ml of P-buffer, filtered through sterile cheesecloth to remove plant debris and centrifuged (10,000 × *g* at 4°C for 15 min). Pellets were resuspended into 1 ml of sterile double distilled water (ddH_2_O) for bacterial DNA extraction for qRT-PCR or plating to selective media. Supernatants were used for phage DNA extraction for qRT-PCR or direct plating to quantify PFU (see below).

### qRT-PCR

Grapevine tissue extracts were assayed for phage and *Xf* by qRT-PCR. The propidium monoazide (PMA) protocol as described by Nocker *et al*. (2006) was used to detect viable cells of *Xf* [23]. Please see the S1 Protocol.

### Phage-resistant *Xf* mutants

To screen for the occurrence of phage-resistant *Xf* mutants *in planta*, isolates were obtained from both for therapeutically and prophylactically treated grapevines at 12 or 9 weeks post-*Xf*-T1 inoculation, respectively. All isolates were confirmed at the species and subspecies level using PCR analysis as described by Hernandez-Martinez *et al*. (2006) [24] and were screened for phage sensitivity by the serial dilution spot assay on overlays and soft agar overlay method as described by Ahern *et al*. (2014) [20]. Additionally, *in vitro* phage-resistant mutants of *Xf*-T1 were selected for using soft agar overlay method as described by Ahern *et al*. (2014) [20] with modifications. Briefly, 100μl of *Xf*-T1 (1 × 10^8^ CFU/ml) and 100μl (1 × 10^8^ PFU/ml) of each cocktail phage were individually mixed in 7 ml of PW-MA soft agar and poured on PW-MA and incubated at 28°C. Plates were evaluated for growth of single colonies for up to 10 d. Colonies were streak purified three times and confirmed at the species and subspecies level using PCR analysis as described above. Phage sensitivity was determined by the serial dilution spot assay on overlays and soft agar overlay method as described by Ahern *et al*. (2014) [20].

### Screening of twitching motility

Twitching motility of *Xf*-T1 and derivatives was examined as previously described by Ahern *et al*. (2014) [20].

### Movement and pathogenicity of phage-resistant *Xf* mutants

Phage-resistant mutants were evaluated for ability to move through grapevines and cause PD symptoms. 15 grapevines each were inoculated with *Xf*-T1 or phage-resistant *Xf*-T1 mutants as described above. Segments from triplicate grapevines were assayed at week 4, 6, 8, and 10 post-inoculation and assayed by qRT-PCR as described above. Grapevines were evaluated for disease symptoms weekly for 12 weeks.

### Statistical analysis

The mean and standard deviation (SD) were calculated from the levels of phages Sano, Salvo Prado, and Paz, and/or *Xf*-T1 obtained from triplicate grapevine samples. ANOVAs were performed using split-plot design to test for significant variation between vine segments across treatments for three independent experiments. Tukey’s Honestly Significant Difference (HSD) test and Student’s *t* test were used for pairwise comparisons in order to test for significant differences between PFUs of grapevines treated only with cocktail verses therapeutically treated grapevines with cocktail or PFUs of grapevines treated only with cocktail verses grapevines prophylactically treated with cocktail. The same tests were used to assess the significant difference between CFUs of grapevines inoculated only with *Xf*-T1verses grapevines treated therapeutically with cocktail or CFUs of grapevines inoculated only with *Xf*-T1 verses grapevines prophylactically treated with cocktail. Significant differences are noted by asterisks (*) in figures. ANOVAs and HSD were performed using Statistix 9.0 (http://www.statistix.com/). Student’s *t* test was performed using R-software version 2.15.3 (http://www.r-project.org).

## Results

### Phage host range and SSR analysis

The host range of *Xf* phages Sano, Salvo, Prado and Paz [20] was evaluated using a collection of 10 Texas and 40 California *Xf* isolates representative of 20 SSR typing groups (S1 and S2 Tables). There was no observed correlation between phage sensitivity and SSR groups. However, phages Sano, Salvo, Prado and Paz formed plaques on 25, 46, 48 and 31 of the 50 *Xf* isolates and on 11, 20, 20 and 16 SSR groups, respectively. The observed overlap indicated that the phages comprising the cocktail had broad host range activity individually and collectively. The four cocktail phages were also able to form plaques on three *X*. *fastidiosa* isolates from coffee plants (*Coffee arabica*) exhibiting coffee leaf scorch symptoms (S1 Table) and on *X*. *fastidiosa* strains Ann-1 and Dixon causing oleander and almond leaf scorch, respectively [20].

### Disease development and movement of *Xf* in grapevines

**Fig 2:**
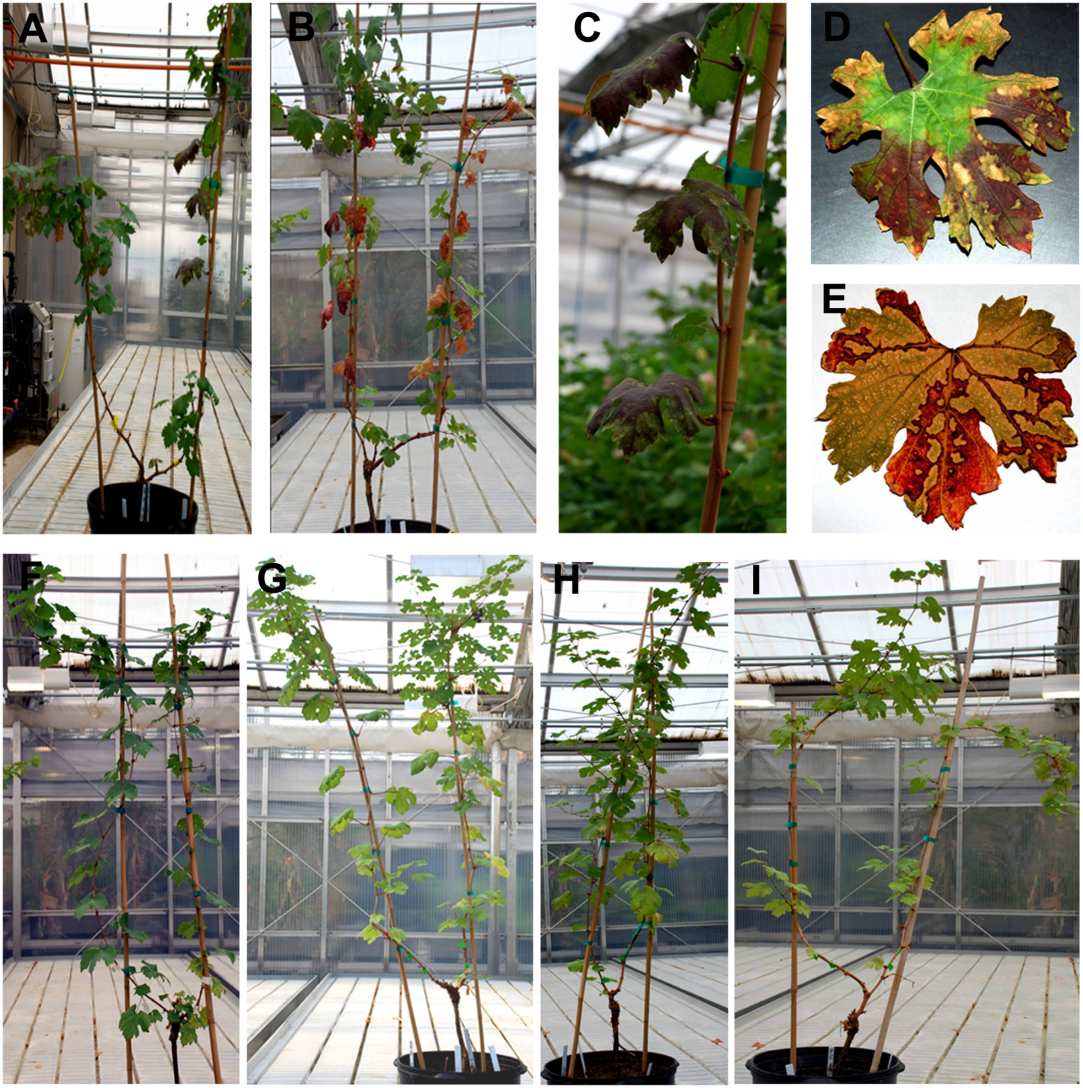
Symptoms of *Xf* in phage non-treated and phage-treated grapevines. (A,B) *Xf* disease control grapevines inoculated with *Xf*-T1. Symptoms at week 8 (A) and 12 (B) post-pathogen inoculation. (C–E) Grapevine leaves exhibiting PD symptoms. (F) Control grapevine inoculated with P-buffer at week 8 (no symptoms). (G) Control grapevine treated only with cocktail phage at week 12 (no symptoms). (H,I) *Xf*-T1 inoculated and phage cocktail treated grapevines. Symptoms at week 8 (H) and week 12 (I) post-pathogen inoculation.

**Fig 3:**
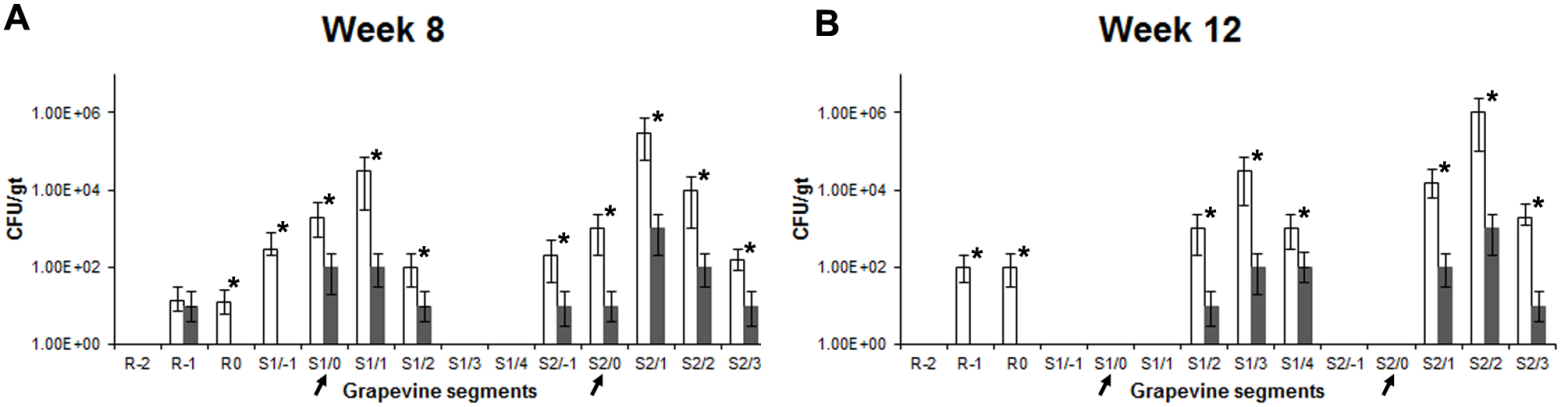
Disease development, movement of *Xf* and therapeutic efficacy of phage cocktail. (A,B) Quantitative levels of *Xf*-T1 at week 8 (A) and 12 (B) post-inoculation of grapevines with only *Xf*-T1 (White bars) (symptoms Fig 2A and 2B, respectively) or *Xf*-T1 and treated with phage cocktail at week 3 post-*Xf*-T1 inoculation (Grey bars) (symptoms Fig 2H and 2I, respectively). POI (indicated with arrows) and grapevine segments numbered as POI (0), below (-) or above (+) in ~13 cm segments. Root divided into three segments and numbered as R0, R-1, or R-2. Segments of similar proximal distance from the graft point from triplicate grapevines were assayed using qRT-PCR to determine the mean CFU/gt ± s.d. Each bar represents s.d. Significant differences are noted by asterisks (*). qRT-PCR line plots obtained for *Xf*-T1 had R^2^ values of greater than 0.9 and efficiencies of 157%.

Characteristic leaf scorching symptoms were first observed on individual leaves of *Xf*-T1 inoculated, non-cocktail treated, grapevines at week 3–4. The leaf symptoms further developed into typical banding pattern of dark red to orange (Fig 2A–2E). Grapevines inoculated with only *Xf*-T1 exhibited full grapevine PD symptoms by week 8 and 12 post-inoculation (Fig 2A and 2B). Control grapevines inoculated with P-buffer [20] did not exhibit PD symptoms (Fig 2F). qRT-PCR of extracts from duplicate cordons representing triplicate samples of the grapevines inoculated with *Xf*-T1 showed movement of the pathogen throughout the grapevine segments assayed (Fig 3A and 3B, White bars). *Xf*-T1 was detected at the points of inoculation (POI) in cordons (~10^3^ CFU/ gm of tissue (gt)), the lower segments (including root systems) at low concentrations (~10^2^–10^3^ CFU/gt) and at higher levels (maxima ~10^5^ CFU/gt) in the upper segments of the trunks at week 8 post-inoculation (Fig 3A, White bars). At week 12 post-inoculation, *Xf*-T1 was detected in the root systems at low concentrations (~10^2^ CFU/gt) and in upper segments of both cordons at higher concentrations (~10^3^–10^6^ CFU/gt; Fig 3B, White bars). Control grapevines (P-buffer inoculated) were confirmed as *Xf* free by qRT-PCR.

### Phage distribution and persistence in grapevines

**Fig 4:**
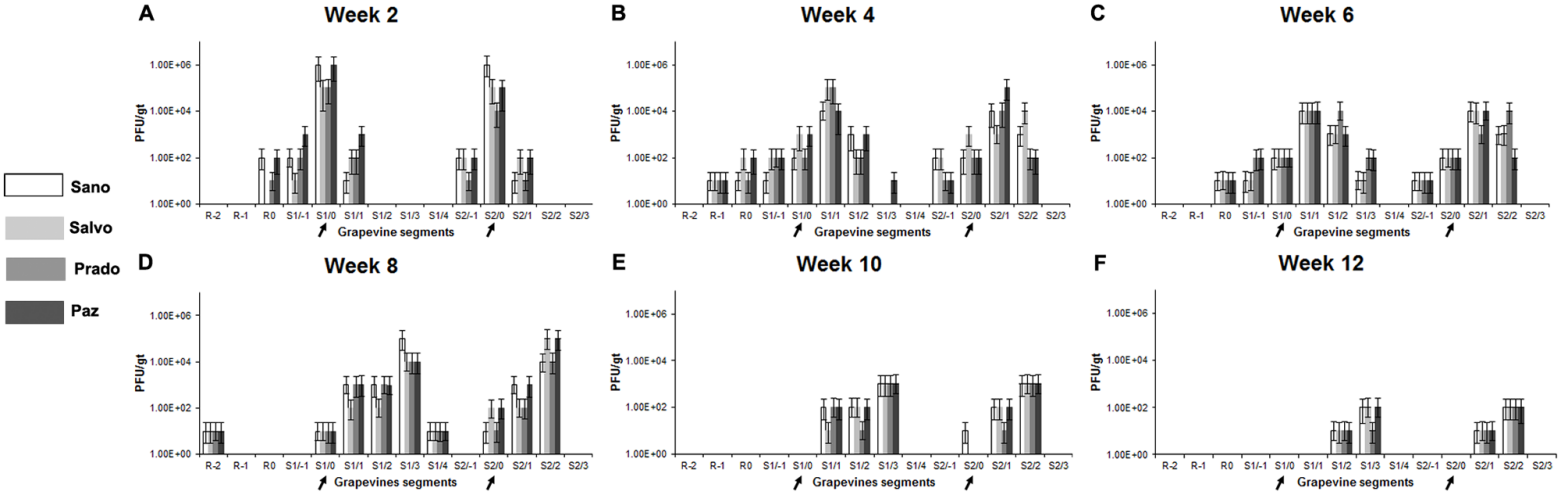
Distribution and persistence of cocktail phages in grapevines. (A–F) Distribution of individual cocktail phage (color coded to left) in grapevines was determined at week 2 (A), 4 (B), 6 (C), 8 (D), 10 (E) and 12 (F) post-inoculation. POI (indicated with arrows) and grapevine segments numbered as POI (0), below (-) or above (+) in ~13 cm segments. Root divided into three segments and numbered as R0, R-1, or R-2. Segments of similar proximal distance from the graft point from triplicate grapevines were assayed using qRT-PCR to determine the mean PFU/gt ± s.d. Each bar represents s.d. Standard qRT-PCR line plots obtained for cocktail phage Sano, Salvo, Prado and Paz had R^2^ values of greater than 0.9 and efficiencies of 129%, 120%, 127% and 123%, respectively.

It was of interest to determine the distribution of cocktail phages in the absence of a host in grapevines, since no prior reports existed. Phage levels in grapevines treated only with cocktail (three grapevines with two cordons each) were quantitatively assessed to determine phage distribution over a 12 week period (Fig 4). The highest concentration of cocktail phages was detected in segments at the POI of cordons (~10^5^–10^6^ PFU/gt) at week 2 post-treatment, with lower levels detected (~10^2^–10^3^ PFU/gt) in the roots and trunk segments ~12 cm above and below the POI (Fig 4A). During week 4, further distribution of phages was detected at ~25 cm above and below the POI, with detection (maxima ~10^2^ PFU/gt) only at ~38 cm above the POI by week 6 (Fig 4B and 4C). Phage levels reached maximum distribution at week 8 (~50 cm above and below the POI) and then declined during weeks 10–12 in the absence of a host to an average of 10^1^–10^2^ PFU/gt in each cordon (Fig 4D–4F). However, the cocktail phages (~4 × 10^1^ PFU/gt) were still detected in petioles collected at ~25–38 cm above the POI at week 12. Grapevines treated only with cocktail did not exhibit disease symptoms at any time during the test period (Fig 2G), nor were cocktail phages detected by qRT-PCR in P-buffer-inoculated grapevines.

### Therapeutic efficacy

**Fig 5:**
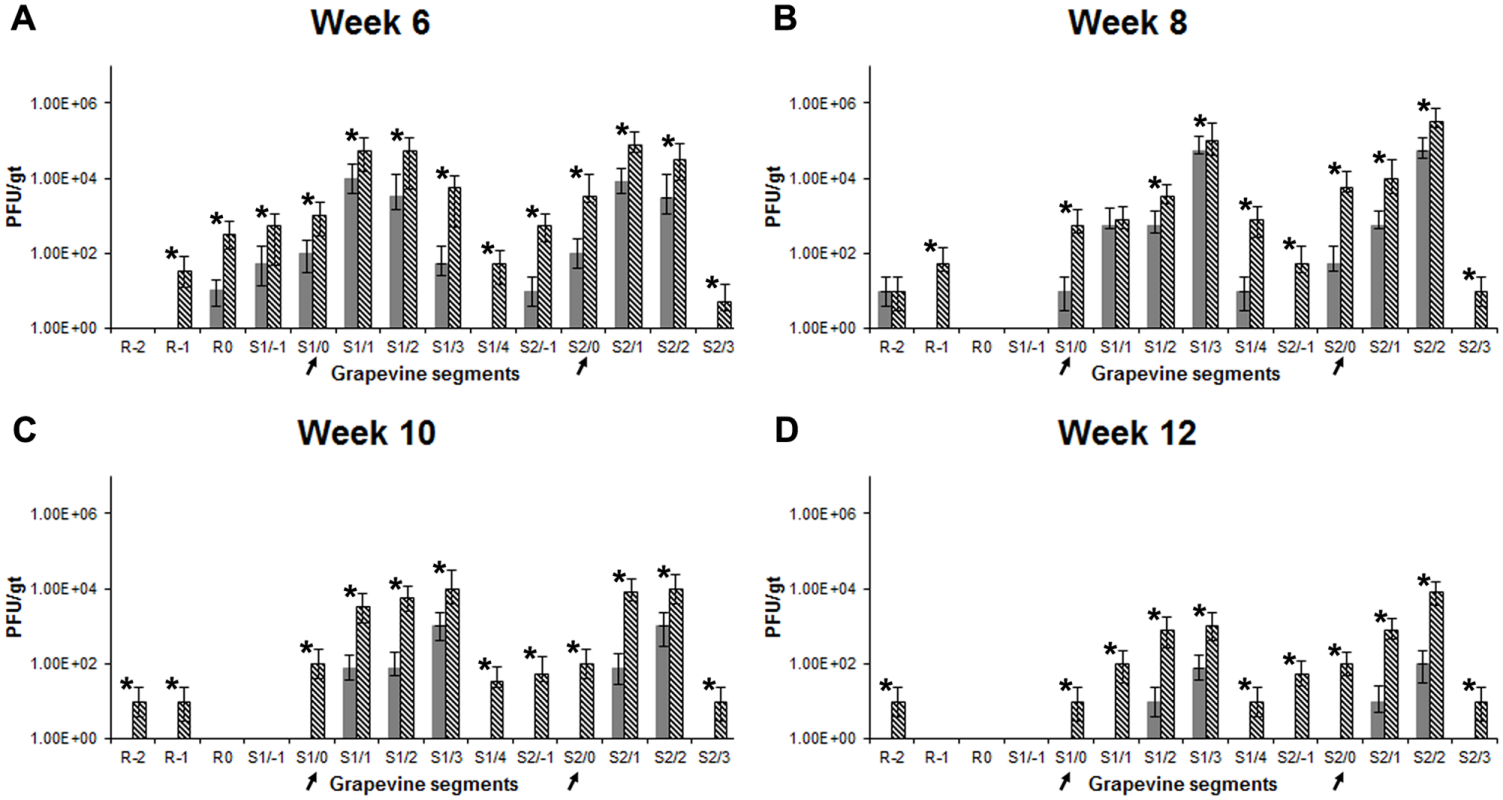
Persistence and replication of cocktail phages in therapeutic study. (A–D) Average quantitative levels of cocktail phages in grapevines treated only with cocktail (Grey bars) and in *Xf*-T1 inoculated and cocktail treated (3 week post-*Xf*-T1 inoculation) grapevines (Crosshatched bars) at week 6 (A), 8 (B), 10 (C) and 12 (D) post-*Xf*-T1 inoculation. POI (indicated with arrows) and grapevine segments numbered as POI (0), below (-) or above (+) in ~13 cm segments. Root divided into three segments and numbered as R0, R-1, or R-2. Segments of similar proximal distance from the graft point from triplicate grapevines were assayed to determine the mean PFU/gt ± s.d. of the four phages in cocktail. Each bar represents s.d. Significant differences are noted by asterisks (*).

To assess potential therapeutic efficacy of the phage cocktail, *Xf*-T1 infected grapevines were treated with phage cocktail at 3 weeks post-pathogen inoculation (S1 Fig, Column 5). At week 8 and 12 (week 5 and 9 post-cocktail treatment, respectively) the level of *Xf*-T1 was significantly lower (10–1000 fold, *P* < 0.05) in treated grapevines (Fig 3A and 3B, Grey bars) as compared to non-treated grapevines (Fig 3A and 3B, White bars). Non-therapeutically treated grapevines showed leaf scorching symptoms as early as week 4, with all grapevines exhibiting typical PD symptoms by week 7–8 (S3 Table, Column 2). In contrast, the cocktail treated grapevines did not further develop symptoms after week 4 (1 week post-cocktail treatment) and were asymptomatic for the duration of the experiment (Fig 2H and 2I, S3 Table, Column 5). The therapeutic effect was correlated with a 10–200 fold (*P* < 0.05) increase in the level of the cocktail phages during week 6 through 12 (Fig 5A–5D, Crosshatched bars) as compared to controls that were treated only with phage cocktail (Fig 5A–5D, Grey bars).

### Prophylactic efficacy

**Fig 6:**
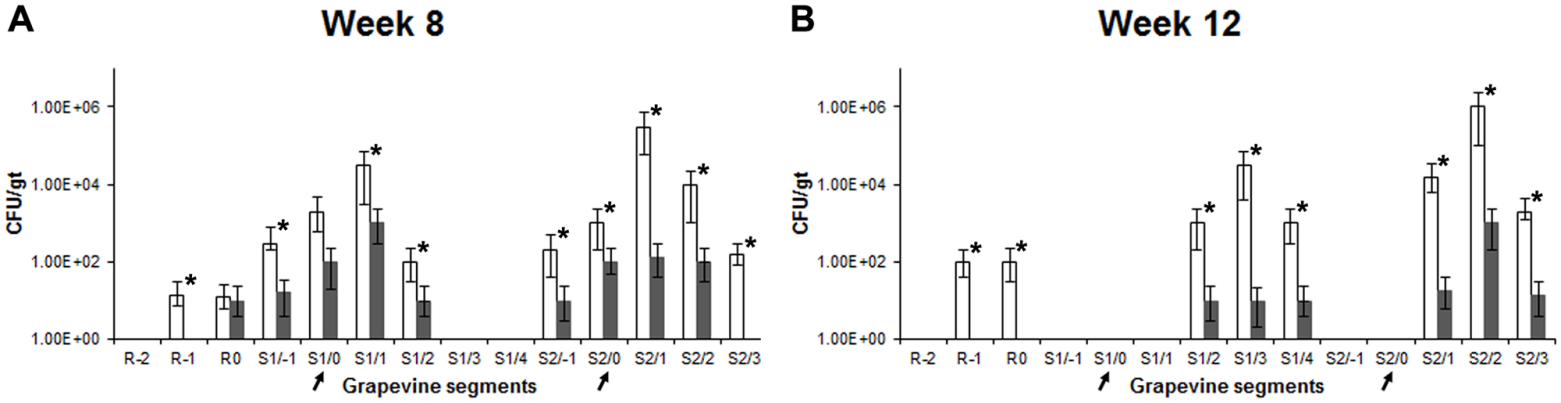
*Xf* levels in non-treated and prophylactically treated grapevines. (A,B) Quantitative levels of *Xf* at week 8 (A) and 12 (B) post-inoculation in grapevines inoculated only with *Xf*-T1 (White bars) or phage cocktail treated and inoculated with *Xf*-T1 at week 3 post-cocktail treatment (Grey bars). POI (indicated with arrows) and grapevine segments numbered as POI (0), below (-) or above (+) in ~13 cm segments. Root divided into three segments and numbered as R0, R-1, or R-2. Segments of similar proximal distance from the graft point from triplicate grapevines were assayed using qRT-PCR to determine the mean CFU/gt ± s.d. Each bar represents s.d. Significant differences are noted by asterisks (*).

Additional studies were conducted with grapevines treated with the phage cocktail and then inoculated with *Xf*-T1 at week 3 post-treatment to determine the prophylactic efficacy of the phage cocktail (S1 Fig, Column 6). In these grapevines, pathogen levels reached a maxima of ~10^3^ CFU/gt (Fig 6A and 6B, Grey bars) in the segments of the grapevines assayed at week 8 and 12 post-phage cocktail treatment, compared to a maxima of ~10^6^ CFU/gt (Fig 6A and 6B, White bars) in non-prophylactically treated grapevines. Moreover, prophylactically treated grapevines showed no PD symptoms at week 8 or 12 (data not shown). As above, the levels of cocktail phages were 10–200 fold higher (*P* < 0.05) in prophylactically treated grapevines compared to cocktail only treated grapevines, and correlated with the prophylactic efficacy (S2A–S2D Fig).

### Phage-resistant *Xf* mutants and twitching motility

At the termination of both therapeutic and prophylactic greenhouse studies at week 12, extracts from grapevine segments from each treatment were plated to determine if surviving *Xf*-T1 isolates were resistant to cocktail phages. Plating of plant extracts from therapeutically or prophylactically treated grapevines yielded an average of 4 × 10^2^ CFU/gt and 3 × 10^2^ CFU/gt, respectively (Figs 3B and 6B, respectively). Representative isolates (20 ea.) from each cordon of each of three grapevines from both therapeutic and prophylactic treatments were confirmed as *Xf* and retained full sensitivity to the cocktail phages (data not shown).

**Fig 7:**
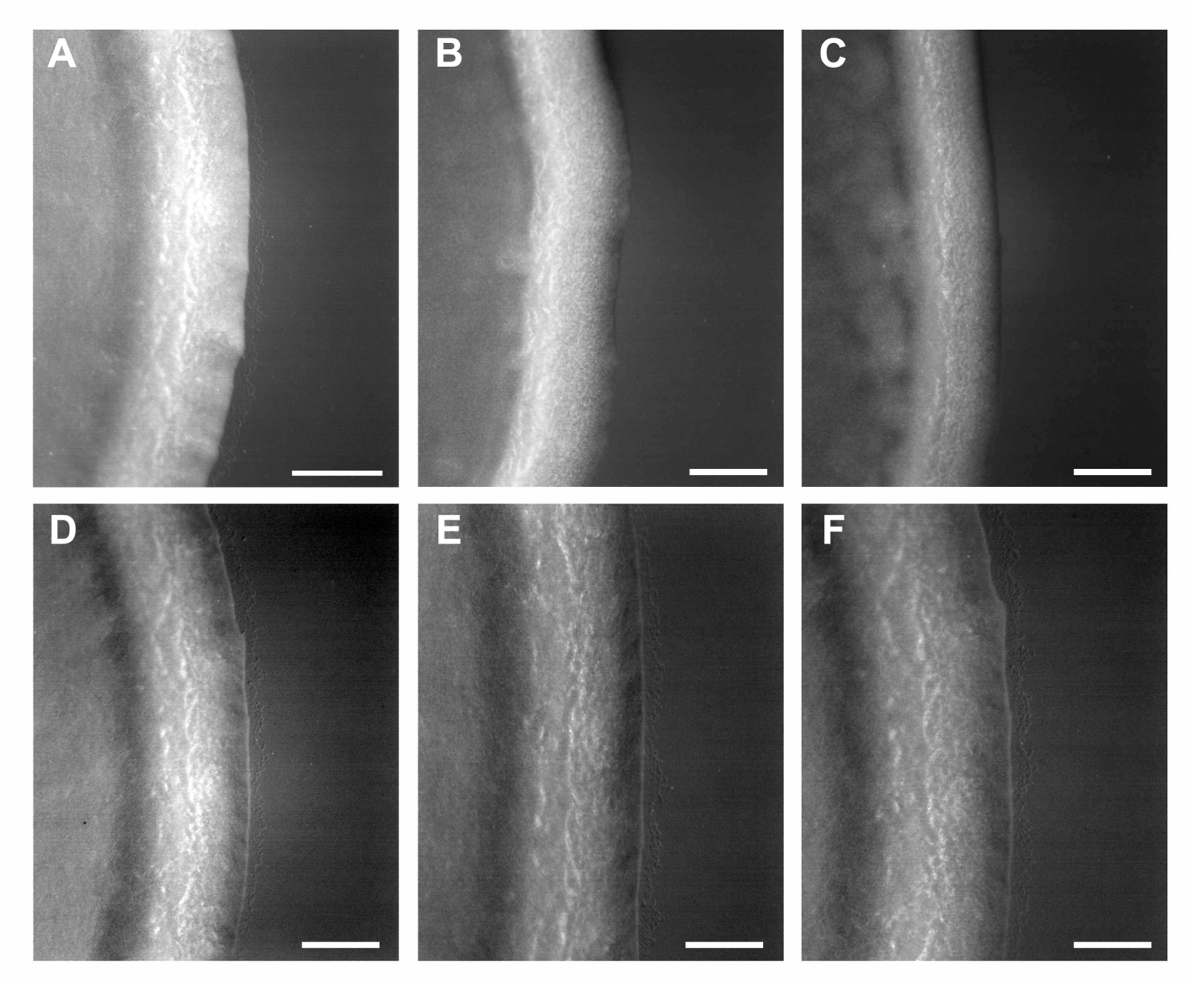
Colony morphology as indicator of twitching motility of wild-type *Xf*-T1 and phage-resistant derivatives. Colonies of the indicated isolates were grown for 5 d (28°C) on the appropriate solid medium modified with 1.2% agar. Colonies with peripheral fringe are twitch positive. (A) wild-type *Xf*-T1 (B) *Xf* Temecula 1Δ*pilA* (C) XF15.51 (D) XF15.7 (E) XF15.11 (F) XF15.37. Bars, 0.1 mm.

Since phage-resistant *Xf* mutants were not found *in planta*, spontaneous *Xf*-T1 mutants resistant to each of the four cocktail phages were selected *in vitro*. All mutants (43 isolates) were confirmed as *Xf* to subspecies level by PCR and were resistant to all four cocktail phages (data not shown). Of eight representative phage-resistant *Xf*-T1 derivatives tested, only one XF15.51 had lost twitching motility (S1 Table and Fig 7). Analysis of the type IV pilus gene clusters of XF15.51 revealed an IS1327 (PD1333; NC_004556.1) insertion in codon 24 of *pilQ* (PD1691; AAO29529.1), which encodes a protein in the outer membrane complex required for the extrusion and retraction of type IV pili [25]. XF15.51 and two of the phage-resistant mutants that retained twitching motility (XF15.7 and XF15.11) exhibited an absolute adsorption defect for all four cocktail phages, whereas the adsorption rate constants for the four phages to the parental *Xf*-T1 were ~4 x 10^−12^ ml cell^-1^ min^-1^, as previously observed (data not shown) [20]. The simplest interpretation of these data is that the four cocktail phages share a requirement for both the type IV pilus, lacking in XF15.1 and an unidentified secondary receptor, defective in the other seven mutants. There was no detectable difference in the growth rate of the phage-resistant mutants as compared to the parental *Xf*-T1 strain in PW broth or solid medium (data not shown).

### Pathogenicity and movement of *Xf* phage-resistant mutants in grapevines

The phage-resistant *Xf* mutants were tested in grapevines to determine pathogenicity and movement. Grapevines inoculated these derivatives (S1 Table) did not develop PD symptoms over a 12 week post-inoculation period, whereas grapevines inoculated with the parental strain (*Xf*-T1) exhibited typical PD symptoms by week 7–8 (data not shown). Triplicate grapevines inoculated with each mutant were segmented and each segment was assayed by qRT-PCR at week 4, 6, 8, and 10 post-inoculation. The non-motile XF15.51 did not exhibit movement in grapevines beyond the POI, since functional type IV pili are required for movement and virulence (data not shown) [26]. In contrast, the other phage-resistant *Xf* that retained twitching motility colonized and showed movement throughout the grapevines (~38 cm above and below the POI) similar to wild type (*Xf*-T1). This suggests the secondary receptor or receptors used by these phages are essential for virulence.

## Discussion

Presently, there are no effective control strategies for PD. The most widely used measure for control of PD is the application of neonicotinoid pesticides that have been reported to adversely affect the winterization of honey bee colonies and contributing to colony collapse disorder [8]. In June 2013, a mass die-off of native pollinators attributed to applications of neonicotinoid insecticides led Oregon state authorities to adopt permanent restrictions on dinotefuran and imidacloprid [27]. Not only do neonicotinoids pose a threat to pollinators, but also they have been found widely in fruits and vegetables and have shown excitatory effects on cultured cerebellar neurons from neonatal rats, suggesting possible neurotoxicity in developing mammalian brains [28,29].

In this study, we demonstrated the therapeutic and prophylactic efficacy for the application of a cocktail composed of four virulent (lytic) *Xf* phages. The phage cocktail reduced the pathogen levels in grapevines and prevented PD symptom development. This is the first report of the application of a phage-based biocontrol strategy for the control of PD and may offer an alternative to the use of broad-acting insecticides for the control of vector-transmitted plant diseases caused by *Xylella* species.

The results show that the phages were able to be distributed in the grapevines after initial introduction, since they were detected in petioles 50 cm above the POI and in root systems 50 cm below the POI in grapevines treated only with phage. In the absence of the host, phage levels declined over a 12 week period (Fig 4). In contrast, in therapeutically and prophylactically treated grapevines phage levels increased 10–200 fold (*P* < 0.05) when a permissive host was present (Fig 5 and S2 Fig). To our knowledge, this is the first report of distribution, persistence and multiplication of phages in a plant vascular system.

A major concern regarding the use of phages as therapeutic agents is the development of phage resistance [18,30,31]. To address this concern, a therapeutic cocktail consisting of four lytic phages exhibiting differential host ranges was used in this study. In multiple *in planta* experiments, we have not detected phage-resistant mutants (data not shown). In fact, we have isolated phage from petioles of grapevines exhibiting PD symptoms [20]. *Xf* is known to persist in grapevines at low levels without causing disease [32], and thus the presence of *Xf* at low levels in the presence of low levels of phage would not be unexpected, since phage-host interactions depend on the concentrations of both viruses and cells [33]. However, phage-resistant derivatives obtained *in vitro* and tested in our system, were all avirulent, irrespective of retention of twitching motility. We have previously reported that the four cocktail phages are lytic and required the presence of type IV pili for adsorption [20]. It was therefore not unexpected to obtain phage-resistant mutants such as XF15.51, with an IS insertion in *pilQ*, and that these mutants would be avirulent *in planta*. However, all other phage-resistant mutants tested were also avirulent, despite the presence of functional type IV pili. This suggests that a secondary receptor is required for phage infection and that this secondary receptor is essential for virulence. Analysis of these mutants to identify the genes responsible is underway.

The implementation of a phage-based biocontrol system composed of a cocktail of lytic phages is a strategy for the treatment or prevention of PD. Furthermore, the target specific nature of the cocktail phages for *Xf* is not harmful to humans, animals, plants or associated beneficial microflora. *In vitro* experiments confirmed that *Xf* subspecies causing almond [20], oleander [20] or coffee leaf scorch were sensitive to the four cocktail phages. The developed phages may offer a treatment for the olive-quick decline syndrome recently reported in Italy that is attributed to *Xylella* [34]. The application of phages as biocontrol agents not only offers the wine industry an effective and environmentally safe method to control PD in grapevines, but also has the potential to control other diseases caused by *Xylella*.

## Supporting Information

S1 Fig Experimental design of the greenhouse trial. (TIF)

S2 Fig Persistence and replication of cocktail phages in prophylactic study. (A–D) Average quantitative levels of cocktail phages in grapevines treated only with cocktail (Grey bars) and cocktail treated and *Xf*-T1 inoculated (3 weeks post-cocktail treatment) grapevines (Crosshatched bars) at week 6 (A), 8 (B), 10 (C) and 12 (D) post-cocktail treatment. POI (indicated with arrows) and grapevine segments numbered as POI (0), below (-) or above (+) in ~13 cm segments. Root divided into three segments and numbered as R0, R-1, or R-2. Segments of similar proximal distance from the graft point from triplicate grapevines were assayed to determine the mean PFU/gt ± s.d. of the four phages in cocktail. Each bar represents s.d. Significant differences are noted by asterisks (*).
(TIF)

S1 Protocol qRT-PCR method. (DOCX)

S1 References (DOCX)

S1 Table Bacterial strains used in this study. (DOCX)

S2 Table Phage sensitivity and SSR groups of *Xf* isolates. (DOCX)

S3 Table PD symptoms development in non-, therapeutically- or prophylactically-treated grapevines. (DOCX)
