# Supplementary figures and images for: Control of Pierce's Disease by Phage
Source: PLoS One. 2015 Jun 24;10(6):e0128902. doi: 10.1371/journal.pone.0128902 (PMC4479439; doi:10.1371/journal.pone.0128902)

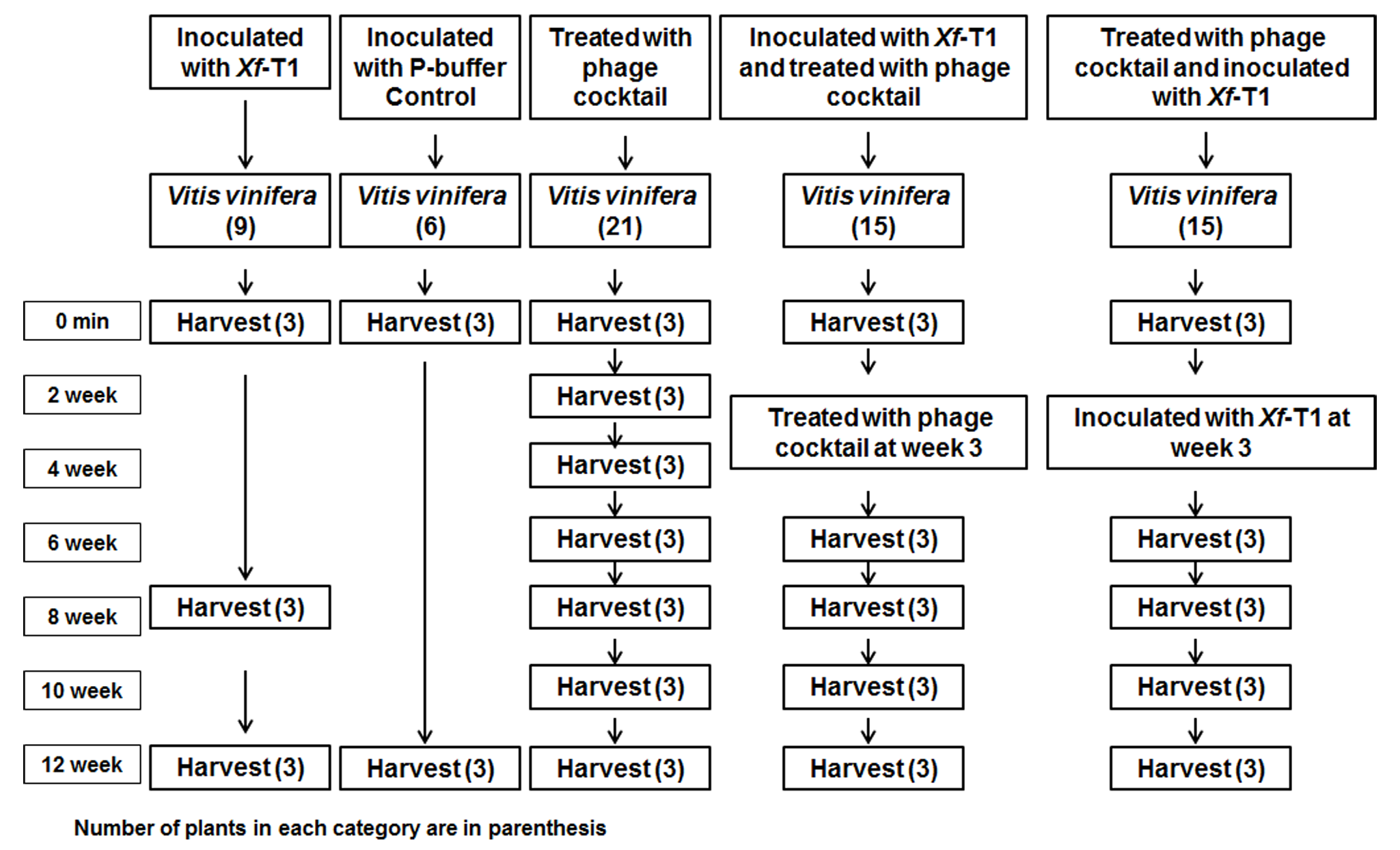

Supplement: S1 Fig — (TIF) [file pone.0128902.s001.tif]

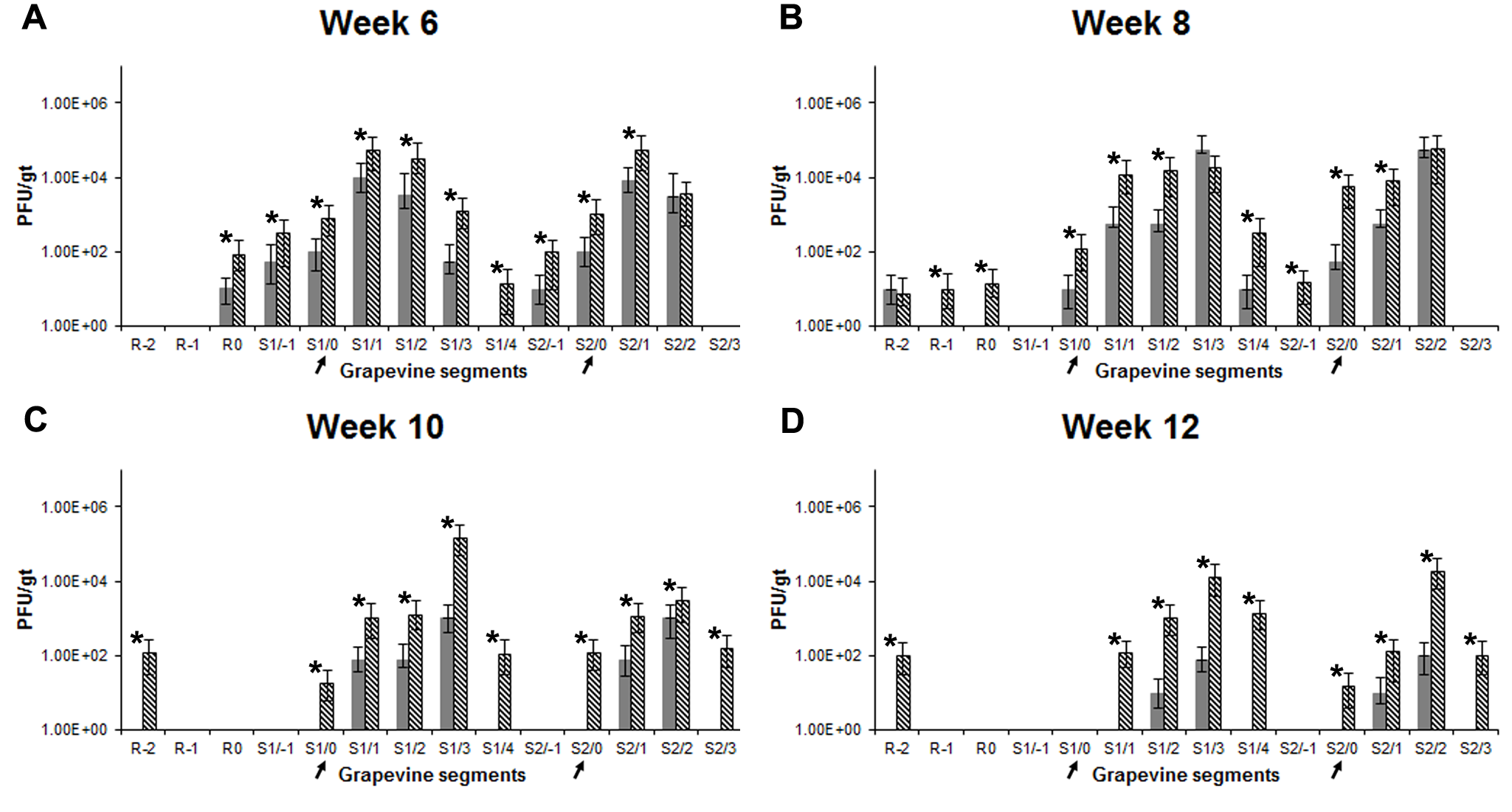

Supplement: S2 Fig — (A–D) Average quantitative levels of cocktail phages in grapevines treated only with cocktail (Grey bars) and cocktail treated and Xf-T1 inoculated (3 weeks post-cocktail treatment) grapevines (Crosshatched bars) at week 6 (A), 8 (B), 10 (C) and 12 (D) post-cocktail treatment. POI (indicated with arrows) and grapevine segments numbered as POI (0), below (-) or above (+) in ~13 cm segments. Root divided into three segments and numbered as R0, R-1, or R-2. Segments of similar proximal distance from the graft point from triplicate grapevines were assayed to determine the mean PFU/gt ± s.d. of the four phages in cocktail. Each bar represents s.d. Significant differences are noted by asterisks (*). (TIF) [file pone.0128902.s002.tif]
