# Supplementary material for: Control of Pierce's Disease by Phage
Source: PLoS One. 2015 Jun 24;10(6):e0128902. doi: 10.1371/journal.pone.0128902 (PMC4479439; doi:10.1371/journal.pone.0128902)
Supplement: S1 Protocol — (DOCX) [file pone.0128902.s003.docx]

Protocol S1. qRT-PCR method.

An aliquot (500μl) of a serial dilution series of *Xf* cell suspensions (10^9^ to 10^1^ CFU/ml) that was confirmed by dilution plating on medium PW-MA, was mixed with PMA (1.25 μl) and incubated in clear microcentrifuge tubes in the dark for 5 min with repeated inversion. Following incubation, the microcentrifuge tubes were placed on ice and exposed to a 650 W halogen light source (Ushio, California, USA) at a distance of 20 cm for 1 min. The tubes were swirled briefly by hand every 15 s and inverted after 30 s of illumination to ensure complete cross-linking of the available DNA and the conversion of free PMA to hydroxylamino propidium. After photo-induced cross-linking, viable cells were collected by centrifugation (12000 × *g* for 2 min at 25 °C) and washed with 500 μl sterile ddH_2_O and resuspended in ddH_2_O water for DNA extraction and dilution plated on medium PW-MA to count viable cells. DNA was extracted from PMA treated cell preparations using a ZR Fungal/Bacterial DNA Miniprep (Zymo Research, California, USA) as per manufacturer’s instructions. Phage DNA was extracted from aliquots (500μl) of a serial dilution series (10^1^ to 10^9^ PFU/ml) from purified phage lysates using Wizard DNA Clean-up system (Promega, Wisconsin, USA) with modifications as described by Summer (2009) [1]. Three replicates of each sample for *Xf* and phages were used to produce the standard curves. Standard curves were constructed by plotting cycle threshold (*Ct*) values generated from qRT-PCR against *Xf* or phage DNA concentrations (Log DNA conc. /µl as determined by A_260_). The efficiency (E) was calculated as follows: E = 10^(-1/slope)^-1 [2]. SYBR-green based qRT-PCR was performed on the 7500 Real-Time PCR System (Applied Biosystems, California, USA) using the *Xf* and phages specific primers reported by Ahern *et al.* [3]. A master mix was made using 10 μl of Express SYBR GreenER SuperMix (Invitrogen, California, USA), 0.4 μl of both primers (at a concentration of 10 μM), 8.56 μl of sterile molecular grade water, 0.04 μl of ROX reference dye (Invitrogen, California, USA) and 1 μl DNA template per reaction. Standardized conditions were used for all reactions with an initial denaturation step of 3 min at 95 °C, followed by 40 cycles of the following parameters: 95 °C for 30 s, 55 °C for 30 s, and 72 °C for 30 s. At the end of PCR, temperature was increased from 72 °C to 99 °C at a rate of 0.5 °C/10 s, and the fluorescence was measured every 10 s. Each DNA sample was analyzed in triplicate. As positive control, DNA was extracted from *Xf*-T1 cells and from phages Prado, Paz, Sano and Salvo using methods described above. *Ct* values, describing the PCR cycle number at which fluorescence rises above the base line, were determined using the software package provided by Applied Biosystems.
