# Supplementary material for: Control of Pierce's Disease by Phage
Source: PLoS One. 2015 Jun 24;10(6):e0128902. doi: 10.1371/journal.pone.0128902 (PMC4479439; doi:10.1371/journal.pone.0128902)
Supplement: S1 References — (DOCX) [file pone.0128902.s004.docx]

**S1 References**

1. Summer EJ. Preparation of a phage DNA fragment library for whole genome shotgun sequencing. Methods Mol Biol. 2009;502: 27-46.

2. Klein D, Janda P, Steinborn R, Muller M, Salmons B, Günzburg WH. Proviral load determination of different feline immunodeficiency virus isolates using real-time polymerase chain reaction: influence of mismatches on quantification. Electrophoresis. 1999;20: 291-299.

3. Ahern SJ, Das M, Bhowmick TS, Young R, Gonzalez CF. Characterization of novel virulent broad-host-range phages of *Xylella fastidiosa* and *Xanthomonas*. J Bacteriol. 2014;196: 459-71.

4. Van Sluys MA, de Oliveira MC, Monteiro-Vitorello CB, Miyaki CY, Furlan LR, , Camargo LE et al. Comparative analyses of the complete genome sequences of Pierce’s disease and citrus variegated chlorosis strains of *Xylella fastidiosa*. J Bacteriol. 2003;185: 1018-1026.

5. Bhattacharyya A, Stilwagen S, Reznik G, Feil H, Feil WS, Anderson I et al. Draft sequencing and comparative genomics of *Xylella fastidiosa* strains reveal novel biological insights. Genome Res. 2002;12: 1556–1563.

6. Montero-Astua M, Hartung JS, Aguilar E, Chacon C, Li W, Albertazzi FJ et al. Genetic diversity of *Xylella fastidiosa* strains from Costa Rica, Sao Paulo, Brazil, and United States. Phytopathology. 2007;97: 1338-1347.

7. Lin H, Civerolo EL, Hu R, Barros S, Francis M, Walker MA. Multilocus Simple Sequence Repeat Markers for Differentiating Strains and Evaluating Genetic Diversity of *Xylella fastidiosa*. Appl Environ Microbiol. 2005;71: 4888-4892.
