# Supplementary material for: Control of Pierce's Disease by Phage
Source: PLoS One. 2015 Jun 24;10(6):e0128902. doi: 10.1371/journal.pone.0128902 (PMC4479439; doi:10.1371/journal.pone.0128902)
Supplement: S1 Table — (DOCX) [file pone.0128902.s005.docx]

Table S1. Bacterial strains used in this study.

| **Strain** | **Genotype or relevant characteristics** | **Reference or source** |
| --- | --- | --- |
| Temecula 1 | *X. fastidiosa subsp. fastidiosa*, Wild-type Pierce’s Disease isolate, ATCC 700964, twitching motility^+^ | 4 |
| XF15-1 | Temecula 1; Δ*pilA*::Km^r^ , twitching motility ^-^ | 3 |
| Ann-1 | *X. fastidiosa* subsp. *sandyi*, oleander isolate, ATCC 700598, twitching motility^+^ | 5 |
| Dixon | *X. fastidiosa* subsp. *multiplex*, almond isolate, ATCC 700965, twitching motility^+^ | 5 |
| Ca-Vc1 | *X. fastidiosa*, coffee isolate | 6 |
| Ca-VIIc2 | *X. fastidiosa*, coffee isolate | 6 |
| Ca-Ic2 | *X. fastidiosa*, coffee isolate | 6 |
| XF15.7 | *X. fastidiosa* Temecula 1 Salvo^a,R^, twitching motility ^+^ | This work |
| XF15.11 | *X. fastidiosa* Temecula 1 Sano^a,R^, twitching motility ^+^ | This work |
| XF15.12 | *X. fastidiosa* Temecula 1 Prado^a,R^, twitching motility ^+^ | This work |
| XF15.16 | *X. fastidiosa* Temecula 1 Sano^a,R^, twitching motility ^+^ | This work |
| XF15.28 | *X. fastidiosa* Temecula 1 Salvo^a,R^, twitching motility ^+^ | This work |
| XF15.37 | *X. fastidiosa* Temecula 1 Paz^a,R^, twitching motility ^+^ | This work |
| XF15.38 | *X. fastidiosa* Temecula 1 Paz^a,R^, twitching motility ^+^ | This work |
| XF15.51 | *X. fastidiosa* Temecula 1 Prado^a,R^, twitching motility ^-^ | This work |
| XF134 - 155, 161 - 163 | *X. fastidiosa* isolate from *V. vinifera*, Santa Clara County, CA | This work |
| XF156 - 160, 164, 165 | *X. fastidiosa* isolate from *V. vinifera*, Sonoma County, CA | This work |
| XF166 - 173 | *X. fastidiosa* isolate from *V. vinifera*, Napa County, CA | This work |
| XF174 - 183 | *X. fastidiosa* isolate from *V. vinifera*, Uvalde County, TX | This work |

Km^r^ = Kanamycin resistant.

^a^ = phage used for selection of phage resistant mutant.

^R^ = Resistant to phages Sano, Salvo, Prado and Paz.

+ = Present

- = Absent
