# Supplementary material for: Control of Pierce's Disease by Phage
Source: PLoS One. 2015 Jun 24;10(6):e0128902. doi: 10.1371/journal.pone.0128902 (PMC4479439; doi:10.1371/journal.pone.0128902)
Supplement: S2 Table — (DOCX) [file pone.0128902.s006.docx]

Table S2. Phage sensitivity and SSR groups of *X. fastidiosa* isolates.

| **Isolate** | **Phage** | | | | **SSR Locus*** | | | | | | | | | | | **SSR Group** |
| --- | --- | --- | --- | --- | --- | --- | --- | --- | --- | --- | --- | --- | --- | --- | --- | --- |
|  | Sano | Salvo | Prado | Paz | OSSR14 | CSSR7 | CSSR10 | CSSR17 | ASSR11 | ASSR12 | GSSR4 | GSSR6 | GSSR14 | GSSR15 | GSSR20 |  |
| Temecula 1 | + | + | + | + | 16** | 14 | 13 | 13 | 9 | 6 | 15 | 8 | 14 | 10 | 4 | G-2 |
| Ann-1 | + | + | + | + | 16 | 15 | 13 | 13 | 9 | 6 | 15 | 8 | 14 | 10 | 4 | G-10 |
| Dixon | + | + | + | + | 17 | 14 | 13 | 13 | 9 | 6 | 15 | 8 | 14 | 10 | 4 | G-13 |
| XF134 | - | + | + | + | 16 | 16 | 13 | 13 | 9 | 6 | 15 | 8 | 14 | 11 | 4 | G-1 |
| XF135 | + | + | + | + | 16 | 14 | 13 | 13 | 9 | 6 | 15 | 8 | 14 | 10 | 4 | G-2 |
| XF136 | - | + | + | - | 16 | 14 | 13 | 13 | 9 | 6 | 15 | 8 | 15 | 10 | 4 | G-3 |
| XF137 | + | + | + | - | 16 | 14 | 13 | 13 | 9 | 6 | 15 | 8 | 14 | 10 | 4 | G-2 |
| XF138 | + | + | + | + | 15 | 14 | 13 | 13 | 9 | 6 | 15 | 9 | 14 | 11 | 4 | G-4 |
| XF139 | + | + | + | + | 16 | 16 | 13 | 13 | 9 | 6 | 15 | 8 | 14 | 10 | 4 | G-5 |
| XF140 | + | + | + | - | 15 | 13 | 13 | 13 | 9 | 6 | 15 | 8 | 14 | 10 | 4 | G-6 |
| XF141 | - | + | + | + | 15 | 14 | 13 | 13 | 9 | 6 | 15 | 8 | 13 | 10 | 4 | G-7 |
| XF142 | + | + | + | + | 15 | 13 | 13 | 13 | 9 | 6 | 15 | 8 | 14 | 10 | 4 | G-6 |
| XF143 | - | + | + | + | 16 | 14 | 13 | 13 | 9 | 6 | 15 | 8 | 13 | 10 | 4 | G-8 |
| XF144 | + | + | + | - | 16 | 13 | 13 | 13 | 9 | 6 | 15 | 8 | 14 | 10 | 4 | G-9 |
| XF145 | - | + | + | + | 16 | 15 | 13 | 13 | 9 | 6 | 15 | 8 | 14 | 10 | 4 | G-10 |
| XF146 | + | + | + | + | 17 | 14 | 13 | 13 | 9 | 6 | 15 | 7 | 15 | 10 | 4 | G-11 |
| XF147 | + | + | + | + | 16 | 15 | 13 | 13 | 9 | 6 | 15 | 8 | 14 | 10 | 4 | G-10 |
| XF148 | - | + | + | + | 16 | 15 | 13 | 13 | 9 | 6 | 15 | 8 | 14 | 12 | 4 | G-12 |
| XF149 | - | + | + | - | 16 | 14 | 13 | 13 | 9 | 6 | 15 | 8 | 14 | 10 | 4 | G-2 |
| XF150 | - | + | + | + | 16 | 14 | 13 | 13 | 9 | 6 | 15 | 8 | 14 | 10 | 4 | G-2 |
| XF151 | - | + | + | - | 16 | 14 | 13 | 13 | 9 | 6 | 15 | 8 | 15 | 10 | 4 | G-3 |
| XF152 | + | + | + | + | 17 | 14 | 13 | 13 | 9 | 6 | 15 | 8 | 14 | 10 | 4 | G-13 |
| XF153 | + | + | + | + | 16 | 14 | 13 | 13 | 9 | 6 | 15 | 8 | 15 | 10 | 4 | G-3 |
| XF154 | - | + | + | + | 17 | 13 | 13 | 13 | 9 | 6 | 15 | 8 | 14 | 10 | 4 | G-14 |
| XF155 | + | + | + | - | 17 | 14 | 13 | 13 | 9 | 6 | 15 | 9 | 15 | 10 | 4 | G-15 |
| XF156 | - | + | + | + | 16 | 13 | 13 | 13 | 9 | 6 | 15 | 7 | 14 | 10 | 4 | G-16 |
| XF157 | - | + | + | + | 17 | 15 | 13 | 13 | 9 | 6 | 15 | 9 | 16 | 9 | 4 | G-17 |
| XF158 | + | + | + | + | 16 | 14 | 13 | 13 | 9 | 6 | 15 | 10 | 16 | 10 | 4 | G-18 |
| XF159 | - | + | + | + | 16 | 14 | 13 | 13 | 9 | 6 | 15 | 8 | 15 | 10 | 4 | G-3 |
| XF160 | + | + | + | - | 16 | 13 | 13 | 13 | 9 | 6 | 15 | 8 | 14 | 10 | 4 | G-9 |
| XF161 | - | + | + | - | 14 | 14 | 13 | 13 | 9 | 6 | 15 | 8 | 14 | 10 | 4 | G-19 |
| XF162 | - | + | + | - | 16 | 14 | 13 | 13 | 9 | 6 | 15 | 8 | 14 | 10 | 4 | G-2 |
| XF163 | + | + | + | + | 16 | 14 | 13 | 13 | 9 | 6 | 15 | 8 | 14 | 10 | 4 | G-2 |
| XF164 | - | + | + | - | 16 | 16 | 13 | 13 | 9 | 6 | 15 | 8 | 14 | 10 | 4 | G-5 |
| XF165 | - | + | + | + | 16 | 15 | 13 | 13 | 9 | 6 | 15 | 8 | 14 | 10 | 4 | G-10 |
| XF166 | - | + | + | - | 16 | 16 | 13 | 13 | 9 | 6 | 15 | 8 | 14 | 10 | 4 | G-5 |
| XF167 | - | + | + | - | 16 | 14 | 13 | 13 | 9 | 6 | 15 | 8 | 13 | 10 | 4 | G-8 |
| XF168 | - | + | + | - | 16 | 13 | 13 | 13 | 9 | 6 | 15 | 8 | 14 | 10 | 4 | G-9 |
| XF169 | - | + | + | - | 16 | 15 | 13 | 13 | 9 | 6 | 15 | 8 | 13 | 8 | 4 | G-20 |
| XF170 | - | + | + | - | 16 | 15 | 13 | 13 | 9 | 6 | 15 | 8 | 13 | 10 | 4 | G-20 |
| XF171 | - | + | + | - | 16 | 16 | 13 | 13 | 9 | 6 | 15 | 8 | 14 | 10 | 4 | G-5 |
| XF172 | - | + | + | - | 16 | 16 | 13 | 13 | 9 | 6 | 15 | 8 | 14 | 10 | 4 | G-5 |
| XF173 | - | + | + | - | 16 | 16 | 13 | 13 | 9 | 6 | 15 | 8 | 14 | 8 | 4 | G-20 |
| XF174 | + | + | - | + | 16 | 13 | 13 | 13 | 9 | 6 | 15 | 8 | 14 | 10 | 4 | G-9 |
| XF175 | + | + | + | + | 16 | 15 | 13 | 13 | 9 | 6 | 15 | 8 | 14 | 10 | 4 | G-10 |
| XF176 | + | + | + | + | 15 | 14 | 13 | 13 | 9 | 6 | 15 | 9 | 14 | 11 | 4 | G-4 |
| XF177 | + | - | + | + | 16 | 14 | 13 | 13 | 9 | 6 | 15 | 8 | 14 | 10 | 4 | G-2 |
| XF178 | + | - | + | + | 15 | 14 | 13 | 13 | 9 | 6 | 15 | 9 | 14 | 11 | 4 | G-4 |
| XF179 | + | - | + | + | 16 | 14 | 13 | 13 | 9 | 6 | 15 | 8 | 14 | 10 | 4 | G-2 |
| XF180 | + | - | + | + | 16 | 16 | 13 | 13 | 9 | 6 | 15 | 8 | 14 | 10 | 4 | G-5 |
| XF181 | + | + | + | + | 16 | 14 | 13 | 13 | 9 | 6 | 15 | 8 | 14 | 10 | 4 | G-2 |
| XF182 | + | + | + | + | 16 | 14 | 13 | 13 | 9 | 6 | 15 | 8 | 15 | 10 | 4 | G-3 |
| XF183 | + | + | - | + | 15 | 14 | 13 | 13 | 9 | 6 | 15 | 9 | 14 | 11 | 4 | G-4 |

(+) = plaques using spot dilution series (10^1^ - 10^4^)

(-) = no plaques on isolate

* Lin *et al.* (2005)^7^

** Number indicates number of alleles identified positively
