# Supplementary material for: Control of Pierce's Disease by Phage
Source: PLoS One. 2015 Jun 24;10(6):e0128902. doi: 10.1371/journal.pone.0128902 (PMC4479439; doi:10.1371/journal.pone.0128902)
Supplement: S3 Table — (DOCX) [file pone.0128902.s007.docx]

Table S3. PD symptoms development in non-, therapeutically- or prophylactically-treated grapevines.

| **Observation for PD symptoms** | **Inoculated with *Xf*-T1**  **(9)*** | **Treated with Phage cocktail**  **(21)*** | **Inoculated with P-Buffer**  **(6)*** | **Inoculated with *Xf*-T1 and treated with phage cocktail**  **(15)*** | **Treated with phage cocktail and inoculated with *Xf*-T1**  **(15)*** |
| --- | --- | --- | --- | --- | --- |
| **Week 0** | 0^§^ | 0 | 0 | 0 | 0 |
| **Week 1** | 0 | 0 | 0 | 0 | 0 |
| **Week 2** | 0 | 0 | 0 | 0 | 0 |
| **Week 3** | 0 | 0 | 0 | 1 | 0 |
| **Week 4** | 1 | 0 | 0 | 2 | 0 |
| **Week 4** | 2 | 0 | 0 | 3 | 0 |
| **Week 5** | 3 | 0 | 0 | 3 | 0 |
| **Week 5** | 3 | 0 | 0 | 3 | 0 |
| **Week 6** | 5 | 0 | 0 | 3 | 0 |
| **Week 6** | 5 | 0 | 0 | 3 | 0 |
| **Week 7** | 6 | 0 | 0 | 3 | 0 |
| **Week 7** | 6 | 0 | 0 | 3 | 0 |
| **Week 8** | 6 | 0 | 0 | 3 | 0 |
| **Week 8** | 6 | 0 | 0 | 3 | 0 |
| **Week 9** | 6 | 0 | 0 | 3 | 0 |
| **Week 9** | 6 | 0 | 0 | 3 | 0 |
| **Week 10** | 6 | 0 | 0 | 3 | 0 |
| **Week 10** | 6 | 0 | 0 | 3 | 0 |
| **Week 12** | 6 | 0 | 0 | 3 | 0 |
| **Week 12** | 6 | 0 | 0 | 3 | 0 |

* Total No. of grapevines in each category is within parentheses. Three vines were harvested at time zero to determine input.

^§^ Number indicated grapevines exhibiting PD symptoms. Grapevines were scored as positive for PD symptoms when a single leaf exhibited symptoms. Grapevines inoculated with only *Xf*-T1 exhibited full vine PD symptoms by week 8 post-inoculation

Note: Red line indicated treatment with phage cocktail or inoculation with *Xf*-T1 at week 3.
